# Predictors of Vancomycin-Resistant *Enterococcus* spp. Intestinal Carriage among High-Risk Patients in University Hospitals in Serbia

**DOI:** 10.3390/antibiotics11091228

**Published:** 2022-09-09

**Authors:** Ana Janjusevic, Ivana Cirkovic, Rajna Minic, Goran Stevanovic, Ivan Soldatovic, Biljana Mihaljevic, Ana Vidovic, Ljiljana Markovic Denic

**Affiliations:** 1 Institute of Virology, Vaccines and Sera “Torlak”, 11152 Belgrade, Serbia; 2 Faculty of Medicine, University of Belgrade, 11000 Belgrade, Serbia; 3 Institute of Microbiology and Immunology, 11000 Belgrade, Serbia; 4 Institute for Medical Research, National Institute of Republic of Serbia, University of Belgrade, 11129 Belgrade, Serbia; 5 Clinic for Infectious and Tropical Diseases, University Clinical Centre of Serbia, 11000 Belgrade, Serbia; 6 Institute of Medical Statistics, 11000 Belgrade, Serbia; 7 Institute of Hematology, University Clinical Centre of Serbia, 11000 Belgrade, Serbia; 8 Institute of Epidemiology, 11129 Belgrade, Serbia

**Keywords:** antibiotic-resistance epidemiology, VRE carriage, risk factors, at-risk inpatients, logistic regression, Serbia, Southeast Europe Region

## Abstract

The predictors of intestinal carriage of vancomycin-resistant *Enterococcus* spp. (VRE) among high-risk patients in the counties of the Southeast Europe Region are insufficiently investigated, yet they could be of key importance in infection control. The aim of the study was to identify risk factors associated with fecal VRE colonization among high-risk inpatients in university hospitals in Serbia. The study comprised 268 inpatients from three university hospitals. Data on patient demographics and clinical characteristics, length of hospital stay, therapy, and procedures were obtained from medical records. Chi-squared tests and univariate and multivariate logistic regressions were performed. Compared to the hemodialysis departments, stay in the geriatric departments, ICUs, and haemato-oncology departments increased the risk for VRE colonization 7.6, 5.4, and 5.5 times, respectively. Compared to inpatients who were hospitalized 48 h before stool sampling for VRE isolation, inpatients hospitalized 3–7, 8–15, and longer than 16 days before sampling had 5.0-, 4.7-, and 6.6-fold higher risk for VRE colonization, respectively. The use of cephalosporins and fluoroquinolones increased the risk for VRE colonization by 2.2 and 1.9 times, respectively. The age ≥ 65 years increased the risk for VRE colonization 2.3 times. In comparison to the University Clinical Centre of Serbia, the hospital stays at Zemun and Zvezdara University Medical Centres were identified as a protector factors. The obtained results could be valuable in predicting the fecal VRE colonization status at patient admission and consequent implementation of infection control measures targeting at-risk inpatients where VRE screening is not routinely performed.

## 1. Introduction

In the last 25 years vancomycin-resistant *Enterococcus* spp. (VRE) has emerged as the one of the most important multidrug-resistant nosocomial pathogens and one of the leading causes of hospital-acquired infections (HAIs) [1]. The inherent characteristics of the species, such as innate and acquired resistance to different groups of antimicrobial drugs and therefore the scarcity of therapeutic options, as well as high endemic capacity that VRE can achieve in the health-care settings [2,3], poses a particular threat to severely ill and immunocompromised inpatients. Therefore, the World Health Organization selected VRE as a pathogen of high priority in its global priority list for the development of new antibiotics [4,5]. Additionally, VRE was established as the member of the group of pathogens bearing the acronym ESKAPE (*Enterococcus faecium*, *Staphylococcus aureus*, *Klebsiella pneumoniae*, *Acinetobacter baumannii*, *Pseudomonas aeruginosa,* and *Enterobacter species*) which represents the most important bacterial causes of hospital infections worldwide, especially notorious for causing invasive infections [6].

The first case of VRE infection emerged in Serbia in 2002 [7], and it was detected in the University Clinical Centre of Serbia (UCCS), the largest university-affiliated tertiary teaching hospital in Belgrade, Serbia, and a main referral center for neighboring countries (e.g., Montenegro, Bosnia and Herzegovina) of Southeast Europe (SEE).

According to the data of the Central Asian and European Surveillance of Antimicrobial Resistance network [8], the frequency of invasive vancomycin-resistant *Enterococcus faecium* (VRE*fm*) isolates in 2020 in Serbia was 60.9%, in comparison to 18.2% which is the average frequency of VRE*fm* in Europe [8]. Hospital-acquired VRE infections could be the tip of the iceberg [9] as VRE colonization often precedes VRE infection [6] and this high frequency may reflect the presence of intrahospital infection and the dissemination of a well-adapted hospital strain in health-care settings.

Prompt identification of patients at-risk for VRE colonization, their isolation, and contact precautions are considered as crucial steps for preventing infections in hospital settings [10], especially in low- and middle-income countries in which microbiological VRE screening upon admission is not regularly performed, due to limited resources [11]. When considering the lack of therapeutic options and the fragile health state in which patients can be admitted to certain hospital wards it is of interest to identify the risk factors for VRE colonization.

Previous studies have identified hospital departments as locations where VRE carriage poses an imminent threat to human health, especially hematology, oncology, hemodialysis, intensive care units (ICUs), geriatrics, liver and bone marrow transplantation units, burns units, neonatology, and acute infectious diseases units [12,13,14,15,16,17,18,19]. Additionally, it has been shown that colonization is higher in university hospitals with a bed capacity exceeding 500 beds [20]. Furthermore, risk factors for VRE colonization such as the age of the patient, application of antimicrobial therapy, length of hospital stay, number of previous hospitalizations in the intensive care unit, diagnostic and therapeutic procedures, surgical intervention, neutropenia, and comorbidities (e.g., diabetes mellitus) have been reported [12,21].

The Republic of Serbia is an upper-middle-income economy based in SEE with a population of about seven million, out of which 1/4 lives in the capital, Belgrade [22]. Our previous study on fecal VRE carriage among at-risk inpatients in university hospitals in Belgrade, Serbia [23], has shown that the prevalence of VRE fecal carriage was 28.7%, which is considerably above the European average and a high portion of multidrug resistance was also detected. Routine VRE carriage screening at hospital admission is not yet introduced in Serbia.

Here, the first comprehensive multicenter analysis of the risk factors associated with fecal VRE colonization among high-risk inpatients in university hospitals in Belgrade, Serbia, is presented. Although VRE screening is an imperative step for preventing VRE dissemination, with this research as an alternative strategy we sought to provide an initial step in creating a helpful tool by targeting at-risk patients.

## 2. Results

### 2.1. Sample Characteristics

Out of 268 patients tested, 77 were intestinal carriers of VRE*fm*. VRE carriage prevalence and phenotypic and genotypic characteristics of isolated VRE stains were presented in a previous paper [23]. Distribution of 77 VRE*fm* and 191 VRE-negative inpatients stratified by demographic and clinical characteristics are shown in Table 1.

We found a significant difference between the VRE carriers and non-VRE carriers in the type of clinical department, the hospital length of stay before the sampling, the history of antimicrobial therapy during the current hospital admission, particularly when it comes to use of cephalosporins and fluoroquinolones, surgical intervention during the current hospital admission, and ICU procedures without a central venous catheter.

The highest frequency of fecal VRE colonization was found in the geriatric wards (42.6%), followed by ICU (40%), haemato-oncology (27.9%), acute infectious diseases (22.7%), and hemodialysis wards (11.7%), which we reported previously [23].

Out of 268 inpatients included in the study, 101 inpatients were recruited from Zemun University Medical Centre (ZmUMC), followed by Zvezdara University Medical Centre (ZvUMC) and UCCS with 86 and 81 inpatients, respectively. The highest frequency of fecal VRE colonization was found in the UCCS with 33.3% (27/81). The frequencies of fecal VRE colonization in ZmUMC (27/101) and ZvUMC (23/86) were the same—26.8%. There was no difference between the VRE carriers and non-VRE carriers in relation to the hospitals included in the study.

According to the length of hospital stay before sampling day 3.9% were VRE carriers among stays of less than 48 h, 29.9% were carriers with stays between 3 and 7 days, 29.9% between 8 and 15 days, and 36.4% were carriers with stays longer than 16 days.

In the VRE colonized group, 66.3% of inpatients received antimicrobial therapy, and 49.4% received a single antimicrobial drug. Furthermore, there is a higher frequency of cephalosporins, fluoroquinolones, and metronidazole use among VRE-carriers compared to the VRE non-carriers. In the VRE-carriers group, the use of cephalosporins was 35%, fluoroquinolone 24%, and metronidazole 23%.

A significantly higher proportion of VRE carriers underwent a surgical intervention and ICU procedures without a central venous catheter since hospital admission.

### 2.2. Univariate Logistic Regression

The results of the univariate logistic regression are presented in Table 2. The predictors of VRE colonization, were found as: age ≥65 years; type of clinical departments; presence of cerebrovascular disease; the hospital length of stay before sampling; surgical intervention during the current admission; antimicrobial treatment during the current admission; administration of cephalosporins; use of fluoroquinolones and metronidazole; ICU procedures without a central venous catheter; and hypoalbuminemia. Based on the analysis of the value of the SE (B) coefficient of the independent variables, we can say that there was no multicollinearity.

In comparison to the hospital stay at UCCS, the hospital stays at ZmUMC and ZvUMC decreased the risk for VRE colonization by 27% each (RR = 0.73, 95% CI 0.385–1.382; RR = 0.73, 95% CI 0.376–1.419). As the level of significance for this association was *p* > 0.1 and this association might be of particular clinical and epidemiological importance, we decided to form a second multivariant model. Therefore, we repeated the multivariant regression analyses in which, in addition to the aforementioned predictors obtained in the univariate analysis, we included the variable “hospitals”.

### 2.3. Multivariate Logistic Regression

The results of multivariate logistic regression are presented in Table 3. Independent predictors for VRE colonization among at-risk inpatients for VRE colonization in Model 1 were hospitalization in clinical wards, hospitalization longer than three days before sampling, and use of cephalosporins and fluoroquinolones. The variable “fluoroquinolones” was not statistically significant in our analysis, however, the lower bound of confidence interval is close to 1, suggesting that is likely that this variable would become significant with increased sample size. Therefore, we left the variable “fluoroquinolones” in the Model 1.

Compared to the hemodialysis departments, a stay in the geriatric departments increased the risk for VRE colonization 6.5 times, a stay in the ICU increased the risk 5 times, and a stay in the haemato-oncology department 4.7 times. Compared to inpatients who were hospitalized 48 h before stool sampling for VRE isolation, inpatients hospitalized 3–7 days before sampling had a 5.6-fold higher risk for VRE colonization, inpatients hospitalized 8–15 days prior to sampling had a 5.5-fold higher risk for VRE colonization, while inpatients hospitalized longer than 16 days prior to sampling had an 8.4-fold higher risk for VRE colonization. The use of cephalosporins increased the risk for VRE colonization 2.2 times and the use of fluoroquinolones 1.8 times.

Independent predictors for VRE colonization among at-risk inpatients for VRE colonization in Model 2 were hospitals, age ≥65 years, hospitalization in clinical wards, hospitalization longer than three days before sampling, and use of cephalosporins and fluoroquinolones. The variable “fluoroquinolones” was also not statistically significant, but we left it in the Model 2 for the previously explained reason.

In comparison to the hospital stay at UCCS, the hospital stays at ZmUMC and ZvUMC decreased the risk for VRE colonization by 74% and 75%, respectively. The age ≥65 years increased the risk for VRE colonization 2.3 times. Compared to the hemodialysis departments, a stay in the geriatric department increased the risk for VRE colonization 7.6 times, a stay in the ICU increased the risk 5.4 times, and a stay in the haemato-oncology department 5.5 times. Compared to inpatients who were hospitalized 48 h before stool sampling for VRE isolation, inpatients hospitalized 3–7 days before sampling had 5-fold higher risk for VRE colonization, inpatients hospitalized 8–15 days prior to sampling had 4.7-fold higher risk for VRE colonization, while inpatients hospitalized longer than 16 days prior to sampling had 6.6-fold higher risk for VRE colonization. The use of cephalosporins increased the risk for VRE colonization 2.2 times and the use of fluoroquinolones 1.9 times.

### 2.4. Evaluation of the Models

**Figure 1:**
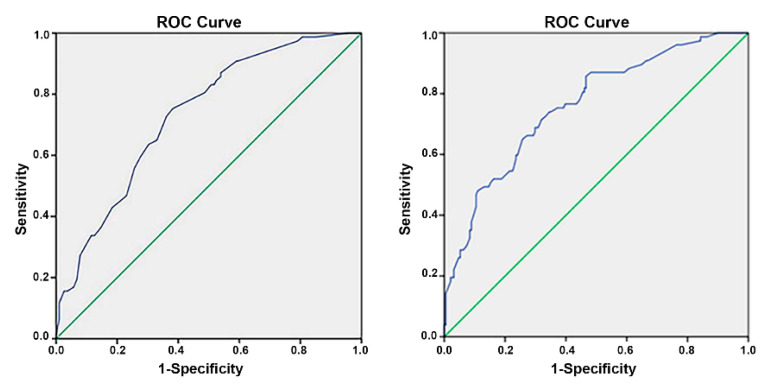
ROC (receiver operating characteristic) curve and AUC (area under the curve) of logistic regression Model 1 (**left**) and Model 2 (**right**).

In Model 1, the relationship between the independent variables and the dependent variable was statistically significant (Omnibus test *p* < 0.001). Model 1 fits the empirical data well. A total of 20.6% of changes in the dependent variable can be explained by changes in independent variables (Nagelkerke R^2^ = 0.206). The Hosmer and Lemeshow goodness-of-fit test resulted in rejection of the null hypothesis, *p* = 0.804, indicating a good match of the observed and predicted risk and the model was well calibrated. The accuracy rate of the logistic regression model was 72,8%. The receiver operating characteristic (ROC) and area under the curve (AUC) of 0.8732 (*p* ≤ 0.001) indicate acceptable discrimination (Figure 1, left).

In Model 2, the relationship between the independent variables and the dependent variable was statistically significant (Omnibus test *p* < 0.001). Model 2 fits the empirical data well. A total of 25.3% of changes in the dependent variable can be explained by changes in independent variables (Nagelkerke R^2^ = 0.253). The Hosmer and Lemeshow goodness-of-fit test resulted in rejection of the null hypothesis, *p* = 0.335, indicating a good match of the observed and predicted risk and the model was well calibrated. The accuracy rate of the logistic regression model was 76.1%. The ROC and AUC of 0.761 (*p* ≤ 0.001) indicate acceptable discrimination (Figure 1, right).

### 2.5. Validation of the Models

In Model 1, the cross-validation was performed on 74% (N = 198) of the original sample size. In the cross-validation analysis, the relationship between the independent variables and the dependent variable was statistically significant (Omnibus test *p* < 0.001), thus, Model 1 fits the empirical data well. A total of 23.2% of changes in the dependent variable can be explained by changes in independent variables (Nagelkerke R^2^ = 0.232). The Hosmer and Lemeshow goodness-of-fit test resulted in a rejection of the null hypothesis, *p* = 0.364, indicating a good match of the observed and predicted risk. The accuracy rate of the logistic regression Model 1 was 74.3%. The ROC and AUC of 0.732 (*p* ≤ 0.001) indicate acceptable discrimination.

In Model 2, the cross-validation was performed on 78.3% (N = 210) of the original sample size. In the cross-validation analysis, the relationship between the independent variables and the dependent variable was statistically significant (Omnibus test *p* < 0.001), thus, Model 2 fits the empirical data well. A total of 26.4% of changes in the dependent variable can be explained by changes in independent variables (Nagelkerke R^2^ = 0.264). The Hosmer and Lemeshow goodness-of-fit test resulted in a rejection of the null hypothesis, *p* = 0.512, indicating a good match of the observed and predicted risk. The accuracy rate of the logistic regression Model 1 was 69.9%. The ROC and AUC of 0.718 (*p* ≤ 0.001) indicate acceptable discrimination.

## 3. Discussion

This study investigated the demographic and clinical predictors of VRE intestinal carriage among high-risk inpatients at university hospitals in Serbia.

Two multivariate regression models were built within our research. In multivariate Model 1, the following variables emerged as the independent predictors for VRE colonization: “hospital departments”, “length of hospital stay before sampling”, “cephalosporins”, and “fluoroquinolones”. In multivariate Model 2, two additional independent predictors for VRE colonization were detected which were “hospitals” and the “age over 65”. Model 2 was a stronger predictor of VRE colonization with R2 0.253 comparing to 0.206 in Model 1.

Our research showed that age over 65 is associated with a 2.3-fold increased risk for VRE colonization. This is similar to results by Karki et al. [24] in a cross-sectional study from a teaching hospital in Melbourne, Australia (OR = 2.19). The retrospective research in a multidisciplinary hospital in Lyon, France, conducted by Djembi et al. [25] also showed that age was associated with an increased likelihood for VRE colonization, although the risk was lower (RR = 1.2) than our report.

In our study, the highest risk for VRE colonization was found in the clinical department for geriatrics. Similar to our findings, Djembi et al. [25] reported a positive association between VRE carriage and hospitalization in a geriatric rehabilitation unit.

A possible explanation for the frequent VRE colonization among elderly and among inpatients in geriatric units could be an increased and prolonged contact with the healthcare system and increased exposure to antimicrobials. Namely, Toh et al. [26] reported increased severity of illness (OR 2.41) as a significant factor associated with prolonged hospital stay among older people. Furthermore, the multivariate logistic regression model by Ojeda-Méndez et. al. [27] found greater morbidity, functional dependence, hypoalbuminemia, anemia, admission to the ICU, and elevated acute phase reactants (CRP) as independently associated with prolonged hospital stay in an acute-geriatric unit. Prolonged hospital stays result in excessive consumption of antibiotics, where only 33.7% of the VRE-carriers in our study did not have a history of recent use of antibiotics. Vancomycin and metronidazole are most often used as therapy for infection caused by Clostridioides difficile in the clinical departments for geriatrics [28], which stimulates VRE colonization [29,30].

In the research of Goossens et al. [31], the frequency of VRE colonization in haemato-oncology and transplantation departments was 31.5%, which is similar to the results obtained in this study. The prevalence of VRE colonization among patients with solid or hematologic malignancy in Europe ranged from 9% to 34%, and in the USA from 13% to 31% [29]. The pooled prevalence of VRE colonization among patients with hematological malignancies was 24% (95% CI, 16%–34%) [29], which is similar to the values detected in this study. The increased risk in the hematology-oncology wards might also be associated with an ongoing exposure to healthcare environments due to prolonged hospital stay and readmission and with more frequent antimicrobial treatment due to febrile neutropenia [32]. Clinical practice guidelines recommend cefepime (4th cephalosporin generation) as the initial antibiotic monotherapy, while fluoroquinolones prophylaxis is recommended for all high-risk hematological patients [33,34]. Cheah et al. (295) created a mathematical model for VRE transmission in hemato-oncology wards and showed that VRE colonization at the aforementioned wards occurred rather endogenously, involving the selective pressure of antimicrobial drugs and the presence of VRE in the environment.

Goossens et al. [31] examined VRE colonization among patients from 13 hospitals in eight European countries, and the highest frequency of VRE colonization (42.3%) was recorded among inpatients in the ICU which is in accordance with our findings. Storms et al. [35] reported the highest rates for ICU admission among older age groups and among persons with underlying medical conditions. Ferreyro et al. [36] reported that cardiovascular disease, chronic obstructive pulmonary disease, and baseline laboratory abnormalities as well as myeloid leukemia, aggressive non-Hodgkin lymphoma, and acute lymphoblastic leukemia were associated with increased risk for ICU admission. In multivariable analysis performed by Vijenthira et al. [37] researchers reported acute leukemia, curative intent chemotherapy, and laboratory-related factors as risk factors for ICU admission among patients with hematologic malignancy. Hawari et al. [38] also found hematologic malignancy, chemotherapy, and advanced cancer as risk factors for ICU admission.

Studies investigating the prevalence of VRE colonization among hemodialysis patients reported values ranging from 1.0% to 7.9% in the USA [39], 13% in Ireland [40], 14.4% in Brazil [41], and 22.0% in Iran [16]. A meta-analysis by Zacharioudakis et al. [42] from 2014 investigated fecal VRE colonization in hemodialysis patients. The overall prevalence of VRE colonization was 6.2% (2.8–10.8%). The frequency of VRE colonization among hemodialysis patients in our study was 11.7% and was most similar to the data from a study in Ireland [40].

Similar to previous research [43,44,45,46,47], this study confirmed that the variable “length of hospital stay before sampling” is independently associated with an increased risk for VRE colonization. In our study, more than 60% of VRE-positive patients stayed in hospital for more than 7 days before the day of sampling, while a third of patients stayed for longer than 16 days before the day of sampling. In the study by Amberpet et al. [45], the risk for VRE colonization among the inpatients who stayed longer in the hospital (8 ± 5.2 days) was 1.2 times higher compared to the inpatients who stayed for a shorter period of time (4.5 ± 2.9 days). In comparison to the aforementioned data, the risks for VRE colonization found in our study are high. Ofori-Asenso et al. [48] found older age and level of comorbidity as factors independently associated with prolonged hospitalization. Additionally, the risk of HAIs increased with the prolonged hospitalization, but at the same time HAIs increase the duration of hospital stay. In addition, inpatients with prolonged hospitalization have a tendency to be more at risk of HAIs due to various comorbidities [49]. Furthermore, HAIs require the use of antimicrobial therapy which leads to the selective pressure of antimicrobial drugs on the gut microbiota and the selection of VRE strains.

Our study showed that the use of cephalosporins increases the risk for VRE colonization by 2.2 times. In the research of Amberpet et al. [45], the use of ceftriaxone (3rd generation cephalosporin) was singled out as a predictor for VRE colonization, increasing the risk for VRE colonization by two times. McEvoy et al. [46] also identified 3rd generation cephalosporins as a risk factor for VRE carriage which increased the risk for VRE colonization more than three times.

The problem with cephalosporins and enterococci is related to the fact that enterococci are innately resistant to cephalosporins. Cephalosporins have a broad spectrum of antimicrobial action, and during therapy with these antimicrobial drugs, microorganisms sensitive to cephalosporins, which make up the dominant microbiome of the gastrointestinal tract, are eliminated and replaced by enterococcus, which then becomes the dominant part of the microbiome of the gastrointestinal tract [50]. Quale et al. [51] showed that restriction of 3rd generation cephalosporins over a period of 6 months leads to a reduction in the frequency of VRE colonization from 47% to 15%. In elderly patients with a urinary tract infection, there are recommendations for empiric antibiotic therapy with a second- or third-generation cephalosporins [52]. Additionally, in elderly patients with pneumonia the treatment should include a fluoroquinolone as monotherapy or the combination of third-generation cephalosporins and a macrolides [53].

McEvoy et al. [46], Karki et al. [54], and Sakka et al. [43] identified fluoroquinolones as risk factors for VRE colonization. Our research showed that the use of fluoroquinolone increases the risk for VRE colonization 1.9 times, which corresponds to the results of McEnoy et al. [46]. The resistance to fluoroquinolones is related to the extent of their consumption. Between 2000 and 2010, a 64% increase in the prescription of fluoroquinolones was recorded worldwide [55]. Fluoroquinolones, especially ciprofloxacin, have a large impact on the microbiome of the gastrointestinal tract, given that high concentrations are reached in the feces during drug excretion. As we mentioned before, parenteral treatment with fluoroquinolones is most often prescribed as a prophylactic therapy or as an initial empiric therapy among hematological patients with febrile neutropenia [33].

A number of studies have shown that the increased use of antimicrobial drugs contributes to the emergence of antimicrobial resistance [56,57]. In the period from 2006 to 2018, an increase in the consumption of antimicrobial drugs was recorded in Serbia: the 3rd generation cephalosporins, 3.2 times; fluoroquinolone 1.6 times; metronidazole 2.2 times; and vancomycin 2 times [58,59,60]. In comparison with other European countries with the highest frequency of invasive VREfm isolates, such as Greece, Ireland, Cyprus, Poland, Hungary, Romania, and Bulgaria, Serbia leads in consumption of cephalosporins, and is second in consumption of fluoroquinolones. The situation is similar with the use of macrolides. Regarding the consumption of vancomycin, Serbia is in 5th place and metronidazole in 4th place among the group of previously listed European countries [3]. Excessive consumption of antimicrobial drugs that are proven inducers of VRE resistance may be one of the potential explanations for the high frequency of VRE colonization obtained in our study.

This research showed that in comparison to the hospital stay at UCCS, the hospital stays at ZmUMC and ZvUMC decreased the risk for VRE colonization by 74% and 75%, respectively. ZmUMC and ZvUMC accommodate mainly patients from the capital, Belgrade, while UCCS is a referral institution for the whole country, where many inpatients are transferred from primary or secondary levels of care including those who are chronically ill or suffer from other comorbidities. Our findings are in line with the fact that VRE colonization of at-risk inpatients for VRE colonization often occurs in urban referral hospitals [14].

A limitation of this study was the cross-sectional study design, so we were not able to determine the timing of acquisition of VRE in inpatients identified as VRE carriers, therefore, inpatients may have acquired VRE in the past, with antibiotic use merely amplifying existing colonization or they may have acquired VRE during prolonged hospitalization.

The identification of risk factors for VRE colonization is the first step in controlling VRE infections; although it does not confer patient protection, it does pinpoint areas of interest for controlling VRE colonization. Additionally, the establishment of known risk factors in an environment of interest enables the design of a grading system, which could ultimately lead to an algorithm for patient triage with the potential to lower prevalence of VRE colonization, at least in countries in which there is no VRE colonization status screening at hospital admission.

## 4. Materials and Methods

### 4.1. Study Design, Setting, and Study Population

This multicenter cross-sectional study comprised 268 inpatients from six hospital departments (geriatrics, ICUs, haemato-oncology, acute infective disease, and hemodialysis) with the capacity of up to 30 beds from three university-affiliated tertiary teaching hospitals: UCCS, ZvUMC, and ZmUMC from Belgrade, Serbia, and spanning the period of 1.5 years (from June 2015 to January 2017) [23].

The study included inpatients of both sexes, aged 18 years and above, who were hospitalized in clinical wards with an increased risk for VRE colonization, from June 2015 to January 2017, and who gave informed consent to participate in the research after the information they received about the research. Patients under the age of 18 were excluded from the research, as well as all patients who, after receiving information about the research, decided not to participate [23].

With a bed capacity of over 3000, and over a million admissions per year, UCCS is the largest tertiary hospital in Serbia. ZvUMC and ZmUMC, with about 800 and 650 beds, respectively, are the second and the third largest referral hospitals in Belgrade, providing a wide range of medical services at the tertiary level. Additionally, ZvUMC has the largest hemodialysis center in Serbia.

Data on patient demographics (age, sex), underlying disease and comorbidities (International Classification of Diseases 10th Revision, ICD-10), hospital treatment (date of admission, transfer from another institution, emergency admission, number of previous hospital treatments, and surgical intervention during admission), antibiotic therapy, and diagnostic and therapeutic procedures were extracted directly from medical records.

Epi info™ 7 (CDC, USA) statistical software was used to calculate the sample size. The calculated target sample size was 227 [23].

### 4.2. Sampling, Isolation, Identification, and Genotyping

Stool samples for VRE testing were collected from 268 inpatients in sterile containers and were processed within 2 h after collection. Chromogenic agar medium (CHROMID^®^VRE, bioMerieux, France) was used for VRE screening. Identification and antimicrobial susceptibility testing were performed using the BD Phoenix™ automated microbiology system (BD, USA). Genotypic identification (*ddl_E. faecium_, ddl_E. faecalis_*) and glycopeptide resistance probing (*van*A, *van*B, *van*C1, *van* C2/C3) were performed using molecular genetic methods [23].

### 4.3. Statistical Analysis

Descriptive statistics, Chi-squared test, and logistic regression were applied in the data analysis using SPSS version 21.0 for Windows (SPSS Inc. Chicago, IL, USA). A univariate logistic regression was used to analyze the association between each risk variable and VRE carriage as the outcome. Based on this model, variables associated with the outcome (VRE carriage) at a level of significance *p* < 0.1 were entered into the final model of the multivariate logistic regression, which was used to compute relative risk (RR) and 95% confidence intervals (95% CI) to assess the independent associations of these variables with the outcome. A stepwise backward method was selected for this analysis. Variables remained in the multivariate logistic regression analysis if they were independently associated with VRE carriage at a significance level of *p* < 0.05. To examine multicollinearity between variables, the standard error (SE) of the B coefficient of the independent variables was used. An SE value greater than 2 suggested that there is multicollinearity between the variables.

Model calibration was assessed using the Hosmer–Lemeshow goodness-of-fit test, discrimination was assessed using the ROC and AUC, and classification accuracy using the Omnibus test and Nagelkerke R Square.

The selected model was further cross-validated (80–20 cross validations) to eliminate model overloading, or in other words, the occurrence of significant, but irrelevant results, due to the large number of predictors used in the analysis.

## 5. Conclusions

This study adds new evidence about risk factors for VRE colonization in Serbia, a middle-income country without routine VRE screening. Independent predictors for VRE colonization among at-risk inpatients were: hospitals; age ≥ 65 years; hospitalization in clinical wards; hospitalization longer than three days before sampling; and use of cephalosporins and fluoroquinolones. The results could be strategically used by stakeholders to modify risk factors that can be controlled (i.e., cephalosporins, fluoroquinolones) and to develop a national grading system that could identify inpatients at high risk for VRE colonization at admission.

## Figures and Tables

**Table 1:** Distribution of vancomycin-resistant *Enterococcus* spp. (VRE) carriers and VRE non-carriers stratified by demographic and clinical characteristics.

Variables	VRE Status				*p*-Value *
	Positive		Negative		
	N (77)	%	N (191)	%	
Gender					0.471
Males	39	50.6	106	55.5	
Females	38	49.4	85	44.5	
Age group (years)					0.057
<65	54	70.1	110	57.6	
≥65	23	29.9	81	42.4	
Hospitals					0.549
Zemun University Medical Centre	27	35.1	74	38.7	
Zvezdara University Medical Centre	23	29.8	63	33.0	
University Clinical Centre of Serbia	27	35.1	54	28.3	
Departments					**0.004**
Geriatrics	23	29.9	31	16.2	
Intensive care units	16	20.8	24	12.6	
Hemato-oncology	22	28.6	57	29.8	
Infectious diseases	10	12.9	34	17.8	
Hemodialysis	6	7.8	45	23.6	
Comorbidities					
Diabetes mellitus	14	18.2	63	32.9	0.978
Hypertension	44	57.1	33	42.9	0.387
Ischemic heart disease	14	18.2	28	14.7	0.473
Heart failure	6	7.8	12	6.3	0.655
Cerebrovascular disease	15	19.5	21	11.0	0.065
Chronic pulmonary disease	8	10.4	11	5.8	0.181
Transfer from another institution					
Yes	5	6.5	9	4.7	0.553
Hospital length of stay before sampling					**0.008**
≤48 h	3	3.9	37	19.4	
3–7 days	23	29.9	52	27.2	
8–15 days	23	29.9	37	19.4	
≥16 days	28	36.4	65	34	
Previous hospital admission					0.544
No	18	23.4	29	15.2	
Yes, during the last 3 months	38	49.4	98	51.3	
Yes, during the previous 3–6 months	2	2.6	9	4.7	
Yes, during the previous 6–12 months	3	3.9	9	4.7	
Yes, more than a year ago	16	20.8	46	24.1	
Surgical intervention during the current admission					**0.023**
Yes	9	11.7	8	4.2	
Surgical intervention in the period of 3 months prior to the current admission	6	7.6	15	7.9	0.987
Yes					
Antibiotics treatment during the current admission					<0.001
No	26	33.8	115	60.2	
Yes, one antimicrobial drug	38	49.4	57	29.8	
Yes, ≥2 antimicrobial drugs	13	16.9	19	9.9	
Groups of antimicrobial drugs used during the current admission					
Beta-lactams without cephalosporins	9	11.7	28	14.7	0.523
Cephalosporins	27	35.1	30	15.7	**<0.001**
Fluoroquinolones	19	24.7	25	13.1	**0.021**
Aminoglycosides	7	9.1	12	6.3	0.418
Vancomycin	10	13.0	17	8.9	0.315
Macrolides and Lincosamides	9	11.7	12	6.3	0.136
Metronidazole	18	23.4	28	14.7	0.087
Other antimicrobial drugs	5	6.5	12	6.3	0.949
Antibiotic treatment in the period of 6 months prior to current admission					
Yes	14	18.2	42	22.1	0.476
Diagnostic–Therapeutic Procedures					
Oncology therapy	22	28.6	57	29.8	0.836
Corticosteroid therapy	17	22.1	20	10.5	0.683
Transfusion	21	27.3	52	27.2	0.756
Urinary catheter	9	11.7	17	8.9	0.485
Central venous catheter	3	3.9	14	7.3	0.297
Hematology procedures	11	14.3	18	9.4	0.246
Intensive care unit procedures without central venous catheter	9	11.7	9	4.7	**0.039**
Endoscopic procedures	9	11.7	27	14.1	0.595
Miscellaneous					
Proton-pump inhibitors treatment	20	26.0	42	22.0	0.484
Probiotics	17	22.1	39	20.4	0.762
*Clostridioides difficile* infection	3	3.9	3	1.6	0.244
Viral infection	6	7.8	18	9.4	0.672
Neutropenia	11	14.3	22	11.5	0.533
Hypoalbuminemia	45	58.4	88	46.1	0.067
Antifungal treatment	2	2.6	3	1.6	0.574

* Chi-square test; bold—statistically significant.

**Table 2:** Univariate logistic regression.

Variables	Univariate Logistic Regression	
	RR (95% CI)	*p* value
Gender		0.471
Males	0.8 (0.484–1.398)	
Females	1.2 (0.715–2.065)	
Age group (years)		
≥65	1.7 (0.981–3.045)	**0.058**
Hospitals		
Zemun University Medical Centre	0.73 (0.385–1.382)	**0.353**
Zvezdara University Medical Centre	0.73 (0.376–1.419)	**0.354**
University Clinical Centre of Serbia (ref)		**/**
Departments		
Geriatrics	5.5 (2.030–15.251)	**0.001**
Hemato-oncology	2.9 (1.082–7.741)	**0.034**
Infectious diseases	2.2 (0.730–6.665)	0.161
Intensive care units	5.0 (1.731–14.447)	**0.003**
Hemodialysis (ref)		/
Comorbidities		
Diabetes mellitus	0.9 (0.499–1.966)	0.978
Hypertension	1.2 (0.742–2.156)	0.387
Ischemic heart disease	1.2 (0.640–2.616)	0.474
Heart failure	1.2 (0.456–3.488)	0.656
Cerebrovascular disease	1.9 (0.950–4.038)	**0.069**
Chronic pulmonary disease	1.8 (0.732–4.916)	0.187
Transfer from another institution		
Yes	1.4 (0.455–4.333)	0.555
Hospital length of stay before sampling		
≤48 h (ref)		/
3–7 days	5.4 (1.525–19.516)	**0.009**
8–15 days	7.6 (2.118–27.755)	**0.002**
≥16 days	5.3 (1.511–18.678)	**0.009**
Previous hospital admission		
No (ref)		/
Yes, during the last 3 m	0.6 (0.311–1.255)	0.186
Yes, during the previous 3–6 m	0.3 (0.069–1.848)	0.22
Yes, during the previous 6–12 m	0.5 (0.128–2.251)	0.395
Yes, more than a year ago	0.5 (0.247–1.270)	0.165
Surgical intervention during the current admission		
Yes	3.0 (1.122–8.166)	**0.029**
Surgical intervention in the period of 3 months prior to the current admission		
Yes	0.9 (0.370–2.658)	0.987
Antibiotics treatment during the current admission		
No (ref)		**/**
1	2.9 (1.632–5.326)	**0.001**
≥2	3.0 (1.328–6.898)	**0.008**
Groups of antimicrobial drugs used during the current admission		
Beta-lactams without cephalosporins	0.7 (0.345–1.719)	0.524
Cephalosporins	2.8 (1.576–5.329)	**0.001**
Fluoroquinolones	2.1 (1.116–4.239)	**0.022**
Aminoglycosides	1.4 (0.564–3.944)	0.420
Vancomycin	1.5 (0.666–3.505)	0.317
Macrolides and Lincosamides	1.9 (0.796–4.896)	0.142
Metronidazole	1.7 (0.915–3.446)	**0.089**
Other antimicrobial drugs	1.0 (0.352–3.046)	0.949
Antibiotic treatment in the period of 6 months prior to the current admission		
Yes	0.7 (0.400–1.535)	0.476
Diagnostic–therapeutic procedures		
Oncology therapy	0.9 (0.525–1.685)	0.836
Corticosteroid therapy	0.9 (0.534–1.711)	0.683 0.756
Transfusion	0.9 (0.551–1.807)	
Urinary catheter	0.7 (0.314–1.736)	0.487
Central venous catheter	1.9 (0.545–6.990)	0.305
Hematology procedures	1.6 (0.718–3.572)	0.249
Intensive care unit procedures without a central venous catheter	2.6 (1.020–7.025)	**0.046**
Endoscopic procedures	0.8 (1.359–1.799)	1.595
Miscellaneous		
Proton-pump inhibitors treatment	1.2 (1.674–2.200)	1.484
Probiotics	1.1 (1.580–2.101)	1.762
*Clostridioides difficile* infection	2.5 (1.501–12.873)	1.260
Viral infection	0.8 (1.310–2.130)	1.672
Neutropenia	1.2 (1.588–2.786)	1.533
Hypoalbuminemia	1.6 (1.964–2.811)	1.068
Antifungal treatment	1.6 (1.274–11.202)	1.578

RR—relative risk; CI–confidence interval; bold—statistically significant; ref—reference category

**Table 3:** Multivariate logistic regression.

Variables	Model 1		Model 2	
	RR (95% CI)	*p* value	RR (95% CI)	*p* value
Age group (years)				
≥65			2.3 (1.039–4.930)	**0.040**
Hospitals				
Zemun University Medical Centre			0.26 (0.096–0.706)	**0.008**
Zvezdara University Medical Centre			0.25 (0.094–0.685)	**0.007**
University Clinical Centre of Serbia (ref)				/
Departments				
Geriatrics	6.5 (2.185–19.747)	**0.001**	7.6 (2.272–25.523)	**0.001**
Hemato-oncology	4.7 (1.599–14.204)	**0.005**	5.5 (1.697–18.042)	**0.005**
Infectious diseases	2.0 (0.580–7.308)	0.264	1.03 (0.244–4.318)	0.972
Intensive care units	5.0 (1.523–16.491)	**0.008**	5.4(1.538–18.853)	**0.008**
Hemodialysis (ref)		/		
Hospital length of stay before sampling				
≤48 h (ref)		/		/
3–7 days	5.5 (1.519–20.388)	**0.010**	5.0 (1.322–18.983)	**0.018**
8–15 days	5.4 (1.450–20.655)	**0.012**	4.7 (1.215–18.128)	**0.025**
≥16 days	8.4 (2.236–31.529)	**0.002**	6.6 (1.687–26.118)	**0.007**
Cephalosporins	2.2 (1.093–4.351)	**0.027**	2.2 (1.114–4.594)	**0.024**
Fluoroquinolones	1.8 (0.865–4.066)	0.111	1.9 (0.846–4.315)	0.119
Nagelkerke R^2^	0.206		0.253	

RR—relative risk; CI—confidence interval; bold—statistically significant; ref—reference category.

## Data Availability

Data supporting the results of this study are not publicly available but can be made available on request to the corresponding author.
